# A Text-Book of Comparative Physiology

**Published:** 1891-03

**Authors:** 


A TEXT-BOOK OF COMPARATIVE PHYSIOLOGY, FOR STUDENTS AND PRACTITIONERS OF COMPARATIVE (VETERINARY) MEDICINE. By WESLEY MILLS, M. A., M. D., D. V. S., Professor of Physiology in the Faculty of Human Medicine and the Faculty of Comparative Medicine and Veterinary Science of McGill University, Montreal; author of a text-book of Animal Physiology, etc., With 476 illustrations. Small octavo; pp. xx.—636. New York: D. Appleton & Co. 1890.

The author is already widely known through his work on Animal Physiology, and therefore needs no introduction to the medical profession of America. This work is designed as a text-book for students in human and more particularly comparative physiology, and fills a want long existing. The study of comparative anatomy and physiology has been too long neglected in our medical curriculum, perhaps, because no suitable text-book was at command. The larger works of Chanveau, Robert Meade Smith, and the author, dealt too largely with the specialties of comparative anatomy to sanction their use in our short medical courses. Where students have not pursued biological studies in colleges before entering upon their medical course, they should be compelled to attend courses on zoology and comparative medicine before trying to master the intricacies of human medicine. It seems that the work before us is well adapted for these studies, especially in physiology.

The author has followed the same systematic course in treating his subjects so characteristic of his larger work, and begins with the consideration of the simplest forms of life, the unicellular organisms. The chapters on General Biology, Reproduction, Development of the Embryo, and Evolution, are treated in a fascinatıng manner, and hold the reader's attention through chapters which are proverbially considered dry and uninteresting.

The chapters on the Blood, Circulation and Digestion, contain the latest researches in these departments, and are profusely illustrated. To the oft-inquired question : Why does not the stomach digest itself during life, the author says : " The secretion of gastric juice runs parallel with the need of it, and is dependent on the introduction of food, its quality, quantity, etc." In other words, if there is nothing to be digested in the stomach, there is no digestion going on owing to lack of a digester. The stories on Respiration, The Nervous System, The Special Senses and Locomotion, are equally well told, and are full of choice sayings and familiar examples, helping to make the text as simple and cogent as possible. The writer also believes that a good illustration is worth two pages of text, for hardly a page that does not contain one or more well-executed engravings.

The publishers have also done their utmost to clothe this work in the best form possible, and have succeeded admirably.

